# The Putative TCP-1 Chaperonin Is an Important Player Involved in Sialic Acid-Dependent Host Cell Invasion by *Toxoplasma gondii*

**DOI:** 10.3389/fmicb.2020.00258

**Published:** 2020-02-21

**Authors:** Na Yang, Mengen Xing, Yingying Ding, Dawei Wang, Xiaogai Guo, Xiaoyu Sang, Jiaqi Li, Chenghuan Li, Yanhu Wang, Ying Feng, Ran Chen, Xinyi Wang, Ning Jiang, Qijun Chen

**Affiliations:** ^1^Key Laboratory of Livestock Infectious Diseases in Northeast China, Ministry of Education, Key Laboratory of Zoonosis, College of Animal Science and Veterinary Medicine, Shenyang Agricultural University, Shenyang, China; ^2^The Research Unit for Pathogenic Mechanisms of Zoonotic Parasites, Chinese Academy of Medical Sciences, Shenyang, China; ^3^College of Food Science and Engineering, Shenyang Agricultural University, Shenyang, China; ^4^College of Basic Sciences, Shenyang Agricultural University, Shenyang, China

**Keywords:** *Toxoplasma gondii*, host cell, invasion, sialic acid receptor, TgTCP-1

## Abstract

Host cell invasion by *Toxoplasma gondii* is crucial for the survival and proliferation of parasite. The process of *T. gondii* tachyzoite invasion requires interaction between parasite proteins and receptors on the surface of host cells. Sialic acid is one of the important receptors for host cell invasion by *T. gondii*. However, the parasite-derived proteins interacting with sialic acid have not been well characterized. In this study, a novel protein named putative TCP-1 chaperonin (TGME49_318410) in *T. gondii* (TgTCP-1) was targeted and characterized. TgTCP-1 protein colocalized with MIC3 protein, which could be secreted from *T. gondii* tachyzoites, and this protein showed a specific binding activity to sialic acid, and DC and Vero cells *in vitro*. The binding of TgTCP-1 protein to DC and Vero cells were inhibited by either pre-incubation with free sialic acid or neuraminidase treatment of the cells. Moreover, a significant reduction of *T. gondii* invasion in Vero cells was observed after pre-incubation of the cells with recombinant TgTCP-1 protein. These results illustrated that TgTCP-1 is an important molecule involved in sialic acid-dependent host cell invasion by *T. gondii*.

## Introduction

*Toxoplasma gondii*, an obligate intracellular protozoan, can infect almost all types of nucleated cells of warm-blooded vertebrates and humans (Dubey, 2010). Successful host cell invasion is vital for proliferation and dissemination of the parasite (Lebrun et al., 2014). However, the molecular mechanism of parasite invasion still remains unclear. There are two known factors in the host cell invasion by *T. gondii*, including parasite-derived proteins and receptors on the host cell surface. It is known that abundant carbohydrate molecules on host cell surface, such as sialic acid, heparan sulfate and chondroitin sulfate, are receptors for *T. gondii* invasion (Monteiro et al., 1998; Carruthers et al., 2000; Baba et al., 2015), but the parasite ligands interacting with these receptors have not been well-characterized.

Sialic acid, as the most abundant amino sugar molecule on cell surface, has been recognized as an important receptor of protozoan parasites. For instance, merozoites of *Plasmodium falciparum* recognize and interact with N-acetylneuraminic acid residues on red blood cell (RBC) for erythrocyte invasion (Malpede et al., 2013), and *Trypanosoma cruzi* can invade macrophage by parasite trans-sialidase transfering sialic-acid residues from host glycoconjugates to parasite mucins (Morrot, 2013). It has been reported that the infection rate of *T. gondii* for the sialic acid-lacking mutant host cells was lower than that for wild type cells (Monteiro et al., 1998). Also, it was observed about 90% reduction of invasion efficiency when N-acetylneuraminic acid (NANA) was used as a competitor or when host cells were treated with neuraminidase (Blumenschein et al., 2007; Friedrich et al., 2010). Therefore, recognition of sialic acids on the host cell surface is critical for efficient invasion of *T. gondii* than other carbohydrates (Baba et al., 2015).

*Toxoplasma gondii* tachyzoite invasion is a multistep process requiring variety of parasites-derived proteins, including surface antigens (SAGs), microneme proteins (MICs), rhoptry proteins (ROPs), dense granule antigens (GRAs), actin-myosin motor and rhomboid proteins (ROMs) (Lebrun et al., 2014). It was reported that TgMIC1 and TgMIC13 could bind to sialic acid on the host cell surface to mediate *T. gondii* invasion. However, the parasite-derived proteins interacting with sialic acid have not been well characterized (Friedrich et al., 2010). We have just completed a sialic acid binding proteome of *T. gondii* RH strain, and several proteins interacted with this receptor have been identified (Xing M. et al., unpublished). In the present study, a novel protein named putative TgTCP-1 chaperonin encoded by TGME49_318410, was systematically characterized for its interaction with sialic acid receptor on host cell surface during invasion.

## Materials and Methods

### Animals and Ethics Statement

All the animal experiments were approved by the Ethics Committee on Animal Experiments of Laboratory Animal Center of Shenyang Agricultural University, China. The SD rats (about 180 g body weight) and New Zealand white rabbits (female, ≈2 kg per rabbit) for generation of protein-specific antibodies were purchased from Liaoning Changsheng Biotechnology (Benxi, Liaoning, China).

### Parasite

*Toxoplasma gondii* tachyzoites (RH strain) were obtained by cultivation in African green monkey kidney (Vero) cells. Briefly, parasites were syringed with a 27-gauge needle, and were filtered through a 5.0 μm pore membrane (Millipore, United States) and centrifuged at 2,000 rpm for 10 min.

### Cloning and Sequencing of the TgTCP-1 Gene

Total RNA was extracted from *T. gondii* tachyzoites (1 × 10^7^) with the Biozol reagent (Bioer, Hangzhou, China). The cDNA was synthesized using Oligo (dT)18 and random 6-mers according to the manufacture’s protocol of cDNA Synthesis Kit (Takara, Dalian, China). The cDNA was used as the template for cloning of TgTCP-1 gene.

The TgTCP-1 gene was amplified by PCR using fast and high-fidelity DNA polymerase (Takara, Dalian, China). The primers were designed based on the CDS sequence of the TgTCP-1 gene (TGME49_318410 in the ToxoDB database) and were as follows: 5′-ATGGTGTCGATTGTCAACGC-3′ (forward primer) and 5′-TCATGCGCCGCGAGACAT-3′ (reverse primer). PCR products were cloned into pEASY-Blunt Simple Cloning Vector (TransGen, Beijing, China) and sequenced. The sequence was analyzed using the software DNAMAN 7 (Lynnon Biosoft).

### Expression and Identification of Recombinant TgTCP-1

The gene fragment coding for TgTCP-1 was cloned into the pGEX-4T-1 and pET-28a vectors, respectively (Invitrogen, Carlsbad, CA, United States), and the recombinant plasmids were transformed into *E. coli* BL21 (DE3) for protein expression, respectively. The GST- and His-tagged fusion TgTCP-1 proteins were purified using the Glutathione Sepharose^TM^ 4B system (GE Healthcare) and the His GraviTrap^TM^ system (GE Healthcare), respectively, according to the manufacturer’s instructions. The purified proteins were verified by SDS-PAGE and Western blotting.

### Preparation and Purification of Anti-TgTCP-1 Antibodies

Two SD rats and two New Zealand white rabbits were immunized subcutaneously with the His-tagged TgTCP-1 fusion proteins in an equal volume of Freund’s complete adjuvant (Sigma-Aldrich, St. Louis, MO, United States) for the first injection. The second and third injections were carried out in 2 and 4 weeks post-primary injection with the His-tag recombinant proteins in an equal volume of Freund’s incomplete adjuvant (Sigma-Aldrich). The anti-TgTCP-1 sera were collected 10 days after the last immunization. Specific IgG was affinity-purified from the immune sera using Protein A Sepharose^TM^ 4 Fast Flow (GE Healthcare).

### Detection of Native TgTCP-1 Protein in *T. gondii* by Western Blotting

*Toxoplasma gondii* tachyzoites were harvested and purified as described above. The purified tachyzoites (1 × 10^7^) were re-suspended in 200 μL of cold PBS with proteinase inhibitors, lysed by 3 freeze-thaw cycles in liquid nitrogen, and then sonicated on ice. The crude lysate was boiled in the 1 × SDS-PAGE loading buffer for 5 min, and resolved by electrophoresis in a 12% (w/v) SDS-PAGE. The separated proteins in the gel were transferred onto polyvinylidene fluoride (PVDF) membranes (Millipore, United States). Membrances were blocked with 5% (w/v) skim milk in PBS for 1 h at 37°C, and then incubated with the rat anti-TgTCP-1 serum and rabbit anti-TgTCP-1 IgG (dilution 1:1,000) for 1 h at 37°C. After washing in PBST (1% Tween-20), the PVDF membrane was incubated with alkaline phosphatase (AP)-conjugated goat anti-rat IgG (dilution 1:20,000, EASYBIO, Beijing, China) for 1 h at 37°C. Finally, the recognized protein by the specific antibody was visualized with enhanced chemiluminescence reagents (BCIP/NET, Beyotime, Shanghai, China).

### Localization of TgTCP-1 in *T. gondii* Tachyzoites by Immunofluorescence Assays (IFA)

The subcellular localization of TgTCP-1 in both extracellular and intracellular parasites was analyzed by IFA as described previously (Cui et al., 2012). For extracellular tachyzoites, freshly released **T. gondii** RH strain tachyzoites were passed through 5 μm filters, centrifuged at 2,000 rpm for 10 min, and resuspended in PBS. The purified tachyzoites were mounted on slides, dried and fixed with 4% formaldehyde for 15 min at room temperature. For intracellular parasites, **T. gondii** tachyzoites were inoculated into Vero cells that attached to coverslips. After 24 h incubation, the coverslips were washed three times in PBS, and fixed with 4% formaldehyde for 15 min. The cells were permeabilized with 0.1% Triton X-100 for 15 min, and blocked with 3% bovine serum albumin (BSA) in PBS for 30 min at 37°C. The rat anti-TgTCP-1 serum and the rabbit anti-MIC3 IgG were incubated as primary antibody (diluted 1:50 in 3% PBS-BSA), respectively. The un-immunized rat serum and a healthy rabbit IgG were used as controls. After 3 washes with PBS, the slides were further incubated with a secondary antibody (Alexa Fluor^®^ 594 conjugated goat anti-rat IgG diluted 1:1000 in PBS, Invitrogen) for 30 min. Afterward, the coverslips were washed with PBS for 5 min and stained with DAPI for 5 min before examination with a fluorescence microscope (Leica DM4B, Wetzlar, Germany).

### Detection of Secreted TgTCP-1 From *T. gondii* Tachyzoites

In order to verify that TgTCP-1 protein can be secreted into extracellular medium during the invasion or proliferation of parasites, purified RH strain tachyzoites (2 × 10^5^) was inoculated into a cell culture and cultivated without serum for 12 h. Meanwhile, serum-free cells without parasites were also cultivated for 12 h as the negative control. The supernatants of the two groups were collected and concentrated, respectively, for Western blotting with the protein-specific antibodies. *T. gondii* tachyzoite lysates were used as the positive control. The rabbit anti-TgTCP-1 IgG were used as a primary antibody.

In addition, detection of excreted or secreted form of TgTCP-1 from free tachyzoites was also performed as previously described (Dogga et al., 2017). Freshly egressed tachyzoites were resuspended in a buffer (5 mM NaCl, 142 mM KCl, 1 mM MgCl2, 2 mM EGTA, 5.6 mM glucose, 25 mM HEPES, pH to 7.2 with KOH) and centrifuged at 1,050 rpm for 5 min. The pellets were resuspended in 100 μL serum-free DMEM medium containing 2% ethanol and incubated for 30 min at 37°C. After the incubation, the parasites were centrifuged at 1,000 *g* for 5 min at 4°C and the supernatant was collected and re-centrifuged at 2,000 *g* for 5 min at 4°C. The supernatant which containing excreted or secreted antigens (ESA) was boiled with SDS loading buffer prior to Western blotting analysis. A specific rat anti-TCP-1 serum was used to detect the secreted TgTCP-1.

### Sialic Acid-Binding Assay

The binding activity of the TgTCP-1 recombinant protein to sialic acid was studied as previously described (Chen et al., 1998). Briefly, the GST-tagged soluble recombinant protein and GST protein in the concentration of 1 μM were mixed with 50 μL sialic acid-agarose and uncoupled agarose, respectively, and incubated for 2 h at 4°C. The mixtures were centrifuged and washed 3 times with cold PBS after incubation. The pellets were mixed with loading buffer for SDS-PAGE and Western blotting, and the GST-specific monoclonal antibody (EASYBIO) to detect the protein specifically bound to the sialic acid on the agarose beads.

### Competitive Sialic Acid-Binding Assay

In this experiment, 1 μM GST-tagged TgTCP-1 fusion proteins were first incubated with different concentrations of 0.001, 0.01, 0.1, 1, 10, 100 mg/mL sialic acid at 4°C for 30 min. Then the protein-sialic acid mixtures were separately added to 50 μL sialic acid-conjugated agarose and further incubated at 4°C for 2 h. After incubation, the beads were precipitated by centrifugation and washed 3 times with cold PBS or PBST. Finally, the beads were mixed with the loading buffer for SDS-PAGE and Western blotting with GST-specific monoclonal antibody (EASYBIO, diluted 1:3000 in 3% PBS-BSA) and AP-conjugated goat anti-rat IgG (EASYBIO) were used as the primary antibody and secondary antibody, respectively, to determine the binding activity of the recombinant proteins with sialic acid.

### Binding of the Recombinant TgTCP-1 Protein With DC and Vero Cells

To investigate the cell-binding activity of the TgTCP-1 with mammalian cells (DC and Vero cells used in this study), Western blotting and immunofluorescence assay (IFA) were performed. For Western blotting assay, cells were mixed with 0.4 μM GST-tagged TgTCP-1 fusion proteins and incubated for 1 h at 4°C as described previously (Baum et al., 2009). GST protein with equal molarity was incubated with cells as a negative control. The cells, after being washed 3 times with PBS, were mixed with the loading buffer and boiled for SDS-PAGE. Western blotting was performed with an anti-GST monoclonal antibody (EASYBIO) as the primary antibody (diluted 1:3000 in 5% PBS-BSA), and detected with the goat anti-mouse IgG (H + L)-AP conjugated (EASYBIO) (diluted 1:5,000 in PBS).

For immunofluorescence analysis (IFA), both DC and Vero cells were gently scraped from cell cultures and centrifuged in 1,000 rpm for 5 min. The cells were pre-incubated with GST-TgTCP-1 for 1 h and washed with PBS for 3 times. After fixation with 4% PFA for 15 min, the anti-GST monoclonal antibody (EASYBIO, diluted 1:250 in 3% PBS-BSA) and Alexa Fluor^®^ 594 conjugated goat anti-mouse IgG (diluted 1:1000 in PBS) (Invitrogen) were added to detect the specific binding of TgTCP-1 fusion protein on the DC and Vero cells, respectively.

### Binding Competition With Soluble Sialic Acid

1 μM GST-tagged TgTCP-1 fusion protein was pre-incubated with different concentrations of 0.001, 0.01, 0.1, 1, 10, 100 mg/mL sialic acid at 37°C for 30 min prior to addition into Vero cell pellets. The mixture of proteins and sialic acids were further incubated with cells at 37°C for 1 h. After incubation, the cells were precipitated by centrifugation and washed 3 times with PBS. Finally, the cell pellets were resuspended with SDS loading buffer for SDS-PAGE and Western blotting, and detected with a GST-specific monoclonal antibody (EASYBIO, diluted 1:3000 in 3% PBS-BSA).

### Evaluation of TgTCP-1 Binding on Neuraminidase Treated Cells

The specific binding of TgTCP-1 protein to the sialic acid receptor on cell surface was further evaluated by neuraminidase treatment of the cells. Briefly, Vero and DC cells were gently scraped from cell cultures and centrifuged at 1,000 rpm for 5 min. The cells were pre-treated with different concentrations of neuraminidase (0, 1, 2, 5, 10, 20 U/mL) (Sigma-Aldrich) in 1 × reaction buffer at 37°C for 3 h. After 3 washes with PBS, the cells were incubated with GST-TgTCP-1 protein (1 μM in PBS) for 1 h at room temperature. After 3 washes with PBS, halve of the cells was mixed with 5 × SDS loading buffer and boiled for SDS-PAGE and Western blotting. A GST-specific monoclonal antibody was used to detect the binding of GST-TgTCP-1 to the cells. For IFA, the rest cells were blocked with 3% BSA for 30 min and an anti-GST monoclonal antibody (EASYBIO) and Alexa-594-conjugated goat anti-mouse antibody (Invitrogen) were incubated as primary antibody and secondary antibody. The fluorescence intensity of host cells was determined with a confocal laser scanning microscope (Leica SP8).

### Invasion Inhibition Assay With Recombinant TgTCP-1

To determine whether the TgTCP-1 protein can inhibit the sialic acid-dependent invasion of the Vero cells by the parasite, the cells were pre-incubated with the recombinant TgTCP-1 protein for 1 h at 37°C, *T. gondii* tachyzoites were added to the cells afterward. Briefly, the GST-tagged TgTCP-1 fusion proteins in concentrations of 0.1, 0.5, 1, 2, 5 μM, respectively, were added to the 6-well cell culture plates and incubated for 1 h at 37°C. Cells only incubated with PBS were used as a blank control. About 2 × 10^5^ tachyzoites were added to each well of the culture plates. Meanwhile, the GST protein was also applied as a negative control. After 4 h of incubation, the cells were washed 6 times with PBS to remove the non-invaded parasites. After continuous cultivation for 12 h at 37°C, the cells were washed 2 times and fixed by methanol, stained with acridine orange. The invasion efficiency of the blank group (incubated with PBS) was set as “100%,” and the invasion efficiency of GST and GST-TgTCP-1 group were calculated relative to the invasion rate of the blank group.

## Results

### Gene Cloning and Analyses of Recombinant and Native TgTCP-1 Proteins

The sequence of the TgTCP-1 protein comprising aa 1–537 is conserved and 100% identical among different strains of *T. gondii* (RH, ME49, GT1, VEG, DOM2, VAND, PRC2, and ARI) as illustrated in the ToxoDB database.^1^

**FIGURE 1:**
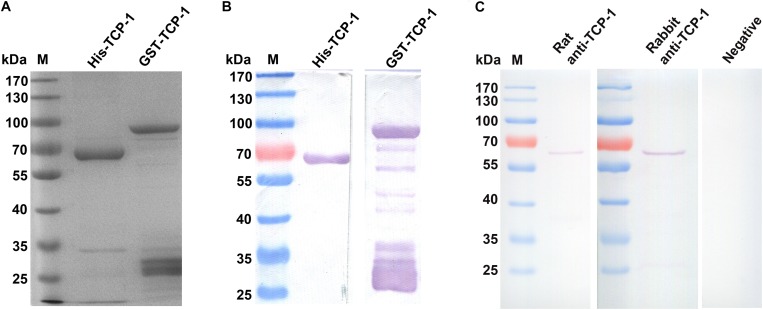
Identification and detection of the recombinant TgTCP-1 proteins. Expression of His- and GST-tagged TgTCP-1 proteins, analyzed by SDS-PAGE **(A)** and Western blotting **(B)**. **(C)** Detection of native TgTCP-1 protein with rat and rabbit specific antibodies by Western blotting.

The His- and GST-tagged fusion proteins were expressed and purified, which had a molecular weight of approximately 60 and 85 kDa in SDS-PAGE (Figure 1A), respectively. The fusion proteins were recognized by the His or GST-specific monoclonal antibody in Western blotting assay (Figure 1B).

The endogenous TgTCP-1 protein with ∼60 kDa in molecular weight in the *T. gondii* tachyzoite lysate was detected by the rat and rabbit anti-TgTCP-1 specific antibody (Figure 1C) in Western blotting assays, whereas the pre-immune sera had no any reaction with the parasite protein.

### TgTCP-1 Was Co-localized With the MIC Proteins in *T. gondii* Tachyzoites

**FIGURE 2:**
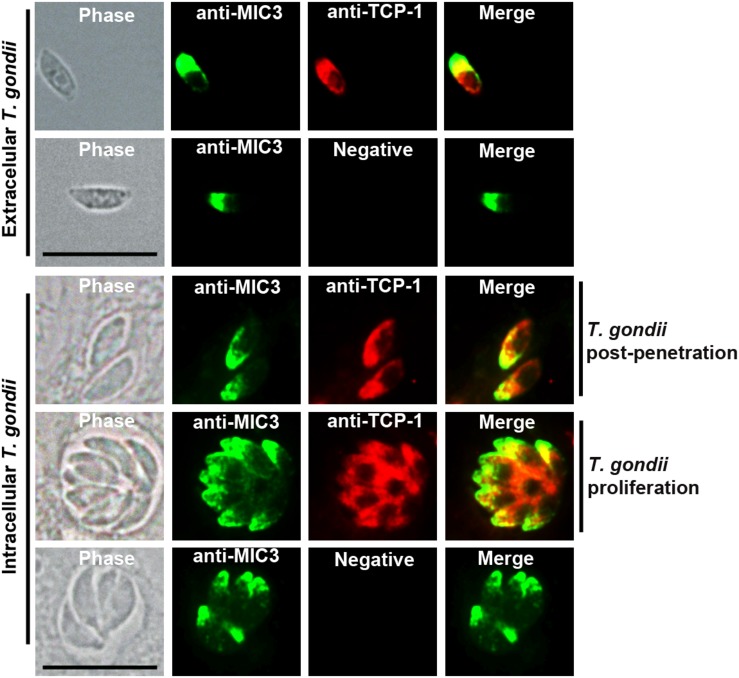
Localization of TgTCP-1 in *T. gondii* tachyzoites by immunofluorescence assay (IFA). The immunofluorescence assay revealed that TgTCP-1 protein was predominantly observed in the apical region (red signal) of both extracellular parasites and in intracellular parasites. Rat anti-TgTCP-1 sera and rabbit anti-MIC3 IgG were used as primary antibodies, the normal rat serum was used as the negative control. Scale bar: 10 μm.

Intracellular and extracellular RH strain tachyzoites were fixed and subjected to IFA using rat anti-TgTCP-1 antibodies to determine the subcellular location of TgTCP-1 in *T. gondii*. The TgTCP-1 protein (red fluorescence) was observed in both intracellular and extracellular tachyzoites, predominantly localized in the apical part of the extracellular parasites colocalized with the TgMIC3 protein (green fluorescence) (Figure 2).

### TgTCP-1 Protein Could Be Secreted Out of the *T. gondii* Parasites

**FIGURE 3:**
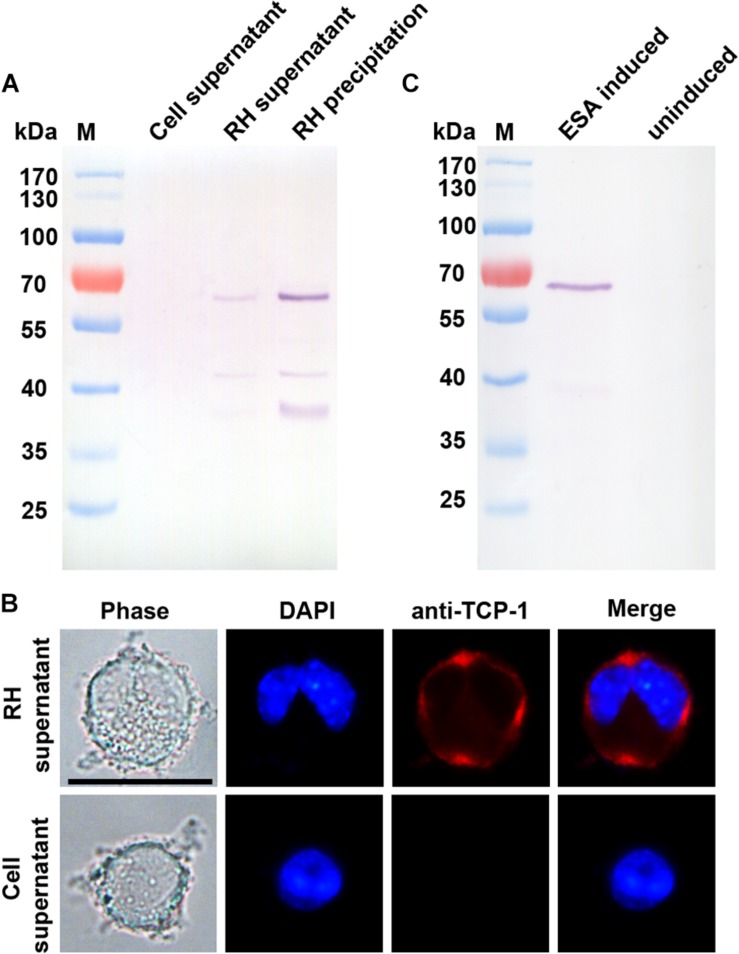
Identification of secreted form of TgTCP-1. **(A)** Determination of native TgTCP-1 protein in the supernatant of parasite-infected cells by Western blotting analysis. Supernatants of RH-free cultures were used as the negative control. **(B)** Detection of TgTCP-1 protein on the DC cells in the culture of *T. gondii* with IFA (red signal). Scale bar: 20 μm. **(C)** TgTCP-1 was detected in the excreted/secreted antigens (ESA) by Western blotting when the pure extracellular parasites were stimulated by 2% Ethanol.

Western blotting analysis showed that the TgTCP-1 protein were secreted into the supernatant of the infected cells, and a specific band (∼60 kDa) was detected with the TgTCP-1-specific antibodies, while it was not present in the supernatant of the cells without *T. gondii* parasites (Figure 3A). In addition, it was found that the binding of the native TgTCP-1 protein to the DC cells was also detected with the protein-specific IgG by immunofluorescent assays (Figure 3B). Meanwhile, TgTCP-1 protein was also detected in the excreted/secreted proteins of extracellular parasites by Western blotting (Figure 3C).

### The Recombinant TgTCP-1 Protein Bound to Sialic Acid

**FIGURE 4:**
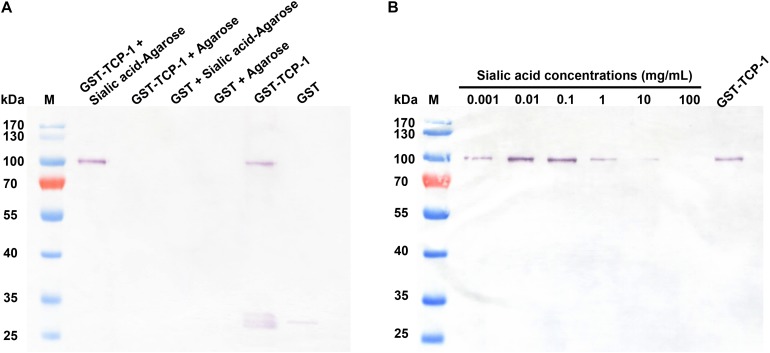
The GST-TgTCP-1 fusion protein specifically bound to sialic acid. **(A)** Evaluation of the binding capacity between GST-TgTCP-1 fusion protein and sialic acid. GST-TgTCP-1 only bound to sialic acid-agarose but not to the agarose beads. GST protein (control) did not bind to both sialic acid-agarose and agarose beads. **(B)** The binding of GST-TgTCP-1 protein with sialic acid-agarose was inhibited by pre-incubation with different concentrations of sialic acids.

In order to determine the sialic acid-binding activity of the TgTCP-1 fusion protein, the GST-tagged TgTCP-1 fusion protein was incubated with sialic acid-agarose and agarose, respectively, and GST tag protein was also incubated with sialic acid. The GST-TgTCP-1 fusion protein bound to sialic acid-agarose but not to agarose, and the GST tag protein did not show any binding to sialic acid-agarose and agarose (Figure 4A).

In sialic acid binding competition assay, the GST-tagged TgTCP-1 fusion protein pre-incubated with different concentrations of sialic acid, and the results showed that the binding of GST-TgTCP-1 to sialic acid-agarose was inhibited by sialic acid in a dose dependent manner (Figure 4B). Both of these assays indicated that TgTCP-1 protein could specifically bind to sialic acid.

### The Recombinant TgTCP-1 Protein Bound to DC and Vero Cells

**FIGURE 5:**
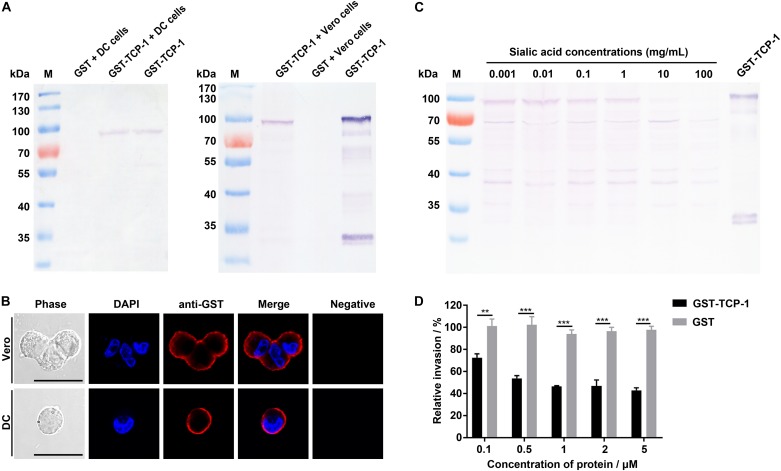
Binding of GST-TgTCP-1 to host cells. **(A)** Western blotting analysis of cell binding *in vitro*. GST-TCP-1 bound to DC and Vero cells, but GST did not bind to the cells. **(B)** The immunofluorescence assay (IFA) illustrated a specific binding of GST-TgTCP-1 to Vero and DC cells detected by a monoclonal anti-GST antibody (red signal). GST did not bind to both cells (negative). Scale bar: 25 μm. **(C)** The binding of GST-TgTCP-1 protein to Vero cells was inhibited by pre-incubation with different concentrations of sialic acids detected in Western blotting. **(D)** Pre-incubation of GST-TgTCP-1 protein with cells significantly inhibited the invasion of *T. gondii*. GST did not inhibit the parasites invasion. Error bars represent the mean ± SD (*n* = 3). ***p < 0.001, **p < 0.01.

The GST-TgTCP-1 protein and GST tag protein were separately incubated with DC and Vero cells, and the adhesion was detected by Western blotting. The results showed that only the GST-TgTCP-1 fusion protein bound to both DC cells and Vero cells, but the GST tag protein did not (Figure 5A). This result was also confirmed by immunofluorescent assays (IFA) (Figure 5B). Thus, the GST-TgTCP-1 fusion protein, but not the GST protein, could specifically bind to DC and Vero cells.

### Sialic Acid Blocked the Binding of TgTCP-1 Protein to Vero Cells

In order to evaluate the specific binding property of TgTCP-1 with sialic acid, the GST- TgTCP-1 fusion protein were pre-incubated with different concentrations of sialic acid prior to interact with Vero cells, and the results showed that the binding of GST-TgTCP-1 to cells was inhibited by sialic acid in a concentration dependent manner (Figure 5C).

### The Recombinant TgTCP-1 Protein Inhibited Parasite Invasion *in vitro*

The sialic acid-dependent invasion blocking effect of the recombinant TgTCP-1 protein was verified by using protein competition assay *in vitro*. The cells pre-incubated with different concentrations of the recombinant TgTCP-1 protein showed resistance to *T. gondii* invasion compared to the control group (Figure 5D).

### Recombinant TgTCP-1 Protein Could Not Bind to Neuraminidase-Treated Cells

**FIGURE 6:**
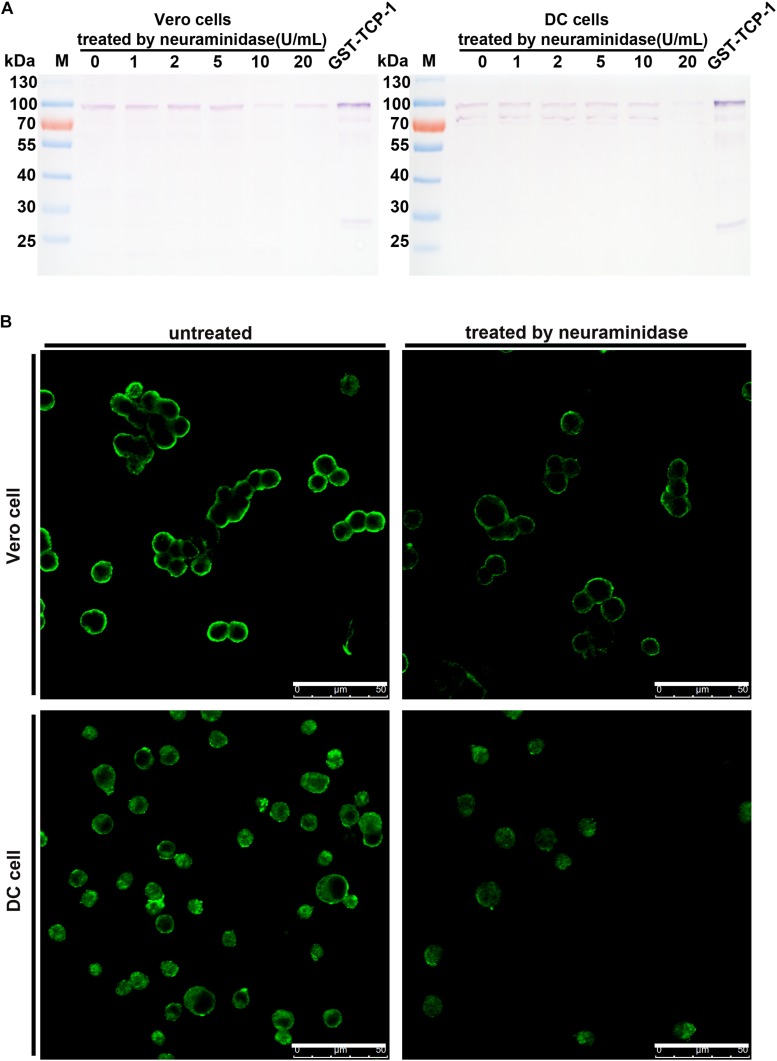
The binding of GST-TCP-1 to host cells was sensitive to neuraminidase treatment. The effect of neuraminidase treatment on the binding of GST-TgTCP-1 to Vero and DC cells was detected by Western blotting **(A)** and IFA **(B)**, the binding of TgTCP-1 to cells was severely weakened when Vero and DC were separately treated by 10 and 20 U/mL neuraminidase. Scalebar: 50 μm.

The specific binding of TgTCP-1 to sialic acid on the DC or Vero cells was further investigated with Western blotting followed neuraminidase treatment of the cells, in which cells were pre-treated with neuraminidase before incubation with recombinant GST-TgTCP-1 protein. The binding of GST-TgTCP-1 fusion protein to Vero cells was severely diminished when the concentration of neuraminidase reached 10 U/ml (Figure 6A). Similarly, the binding of GST-TgTCP-1 fusion protein to DC cells was disappear when the neuraminidase concentration reached 20 U/ml (Figure 6A). Meanwhile, IFA showed that the fluorescence intensity was greatly weakened when Vero cells and DC cells were treated by neuraminidase (Figure 6B). These data confirmed that sialic acid on the cell surface is a specific receptor for TgTCP-1.

## Discussion

Recognition and adhesion of carbohydrate receptors on the host cell surface are critical for cell invasion by apicomplexan parasites (Friedrich et al., 2010). *T. gondii* can actively invade almost all types of nucleated cells including DC cells and Vero cells, and sialic acid residues present on the cell surface can trigger *T. gondii* penetration (Nishikawa et al., 2013). Sialic acid, as the most common component of glycoconjugates on the host cell surface found in all vertebrate tissues, are exploited by a wide range of pathogens including bacteria, viruses and parasites (Friedrich et al., 2010). In recent years, a large number of data have shown that recognition of sialic acids are important in the apicomplexan parasite-host cell interactions (Monteiro et al., 1998; Spadafora et al., 2010; Malpede et al., 2013; Morrot, 2013). Micronemal proteins (MICs), one of the key mediators of cytoadherence and invasion for *T. gondii* (Hager and Carruthers, 2008), and it’s reported that micronemic adhesins, and MIC1 and MIC13 have been reported to build a tight parasite-host interaction through interaction with sialic acids during invasion (Hager and Carruthers, 2008; Friedrich et al., 2010). However, apart from MICs, other invasion-associated ligands remain not to be characterized in *T. gondii*. In this study, we selected a protein, named TgTCP-1 (TGME49_318410), from the sialic acid-binding proteome of *T. gondii* (Xing M. et al., unpublished) for further analysis.

TgTCP-1 protein was expressed in *T. gondii* tachyzoites with a molecular weight of ∼60 kDa. Immunofluorescent assay (IFA) showed that the TgTCP-1 protein was predominantly localized in the apical part of the parasites colocalized with the MIC3 protein (Figure 2). In the intracellular tachyzoites, the TgTCP-1 protein was also mainly localized in the apical region of the parasites (Figure 2). The results of localization suggest that TgTCP-1 protein may be involved in cell invasion of *T. gondii* similar to TgMICs proteins.

In order to verify if TgTCP-1 protein can be secreted into extracellular medium by the parasites, purified RH strain tachyzoites was inoculated into the DC cell culture and cultivated without serum for 12 h, and the TgTCP-1 protein was identified in the supernatant of *T. gondii*-infected cells by Western blotting, and the protein readily bound to the host cell after release (Figures 3A,B). Further, the TgTCP-1 protein was confirmed to be secreted from free *T. gondii* tachyzoites (Figure 3C). These results indicated that the TgTCP-1 is a secreted protein likely for interaction with the host.

To characterize the function of TgTCP-1, we performed sialic acid-binding assay with the fusion protein. The GST-TgTCP-1 protein specifically bound to sialic acid-agarose and did not bind to agarose, and the GST tag protein did not show any binding to sialic acid-agarose and agarose (Figure 4A). Further, the binding of GST-TgTCP-1 fusion proteins to sialic acid-agarose could be blocked by pre-incubation of the fusion protein with sialic acid (Figure 4B). These results indicate that the TgTCP-1 protein can bind specifically to sialic acid.

To further investigate its binding activity to the mammalian cells, GST-TgTCP-1 recombinant proteins were incubated with DC cells and Vero cells, and the data of both Western blotting and IFA showed that the recombinant TgTCP-1 could bind to both DC and Vero cells (Figures 5A,B). The binding was blocked by pre-incubation of the recombinant protein with sialic acid (Figure 5C), which confirmed that the binding of TgTCP-1 to the host cells was through a specific interaction with the sialic acid receptor. Furthermore, the binding of TgTCP-1 protein with sialic acid on the cell surface was verified by cleavage of sialic acids from the surface of DC and Vero cells by neuraminidase (Figures 6A,B). These results suggested that TgTCP-1 protein can specifically bind to sialic acid on host cell surface.

Importantly, the invasion efficiency of *T. gondii* parasites to the host cells pre-incubated with the TgTCP-1 recombinant protein was significantly inhibited, indicating that the interaction of the native TgTCP-1 of parasites with the host cells was blocked by the TgTCP-1 recombinant protein (Figure 5D). These findings collectively suggest that the TgTCP-1 participated in host cell invasion by *T. gondii* through interaction with sialic acid on the surface.

In conclusion, the TgTCP-1 is a protein actively expressed in *T. gondii* tachyzoites, and it is an important molecule involved in sialic acid-dependent host cell invasion by *T. gondii.*

## Data Availability Statement

The datasets generated for this study are available on request to the corresponding author.

## Ethics Statement

The animal study was reviewed and approved by the Ethics Committee on Animal Experiments of Laboratory Animal Center of Shenyang Agricultural University, China.

## Author Contributions

QC designed the study. NY, MX, and YD performed the experiments. QC, NY, MX, YD, NJ, XS, XG, JL, CL, DW, YW, YF, RC, and XW analyzed the data. QC and NY wrote the manuscript.

## Conflict of Interest

The authors declare that the research was conducted in the absence of any commercial or financial relationships that could be construed as a potential conflict of interest.

## Footnotes

1 https://toxodb.org/toxo/
